# Circular RNA circ_RANBP9 exacerbates polycystic ovary syndrome via microRNA-136-5p/*XIAP* axis

**DOI:** 10.1080/21655979.2021.1964157

**Published:** 2021-09-21

**Authors:** Xiaohui Lu, Haijie Gao, Bo Zhu, Guilan Lin

**Affiliations:** a Department of Laboratory Medicine, The First Affiliated Hospital of Xiamen University, Xiamen Key Laboratory of Genetic Testing, Xiamen, Fujian, China; b Department of Reproductive Medicine, Women and Children’s Hospital, School of Medicine, Xiamen University

**Keywords:** Polycystic ovary syndrome, circ_RNABP9, proliferation, apoptosis, miR-136-5p, *XIAP*

## Abstract

Polycystic ovary syndrome (PCOS) is an endocrine disease that affects the health of many women. Circular RNAs (circRNAs) are associated with the occurrence and progression of PCOS. This study aimed to explore the function of circ_RANBP9 in PCOS. First, the circ_RANBP9 level was found to be increased in the plasma of patients with PCOS and ovarian granulosa cells (GCs) using Reverse Transcription-Quantitative Polymerase Chain Reaction (RT-qPCR). In GCs, loss of circ_RANBP9 decelerated proliferation and accelerated apoptosis of KGN and COV434 cells, as determined by MTT assay, colony formation assay, and flow cytometry. Furthermore, bioinformatics analysis showed that circ_RANBP9 and *XIAP* can be targeted by the microRNA, miR-136-5p. Luciferase reporter assay and RNA pull-down assay further verified the interaction between miR-136-5p and circ_RANBP9 or *XIAP*. Importantly, knockdown of circ_RANBP9 suppressed proliferation and promoted apoptosis of KGN and COV434 cells, whereas inhibition of miR-136-5p reversed these effects. Additionally, *XIAP* abolished the repression of proliferation and acceleration of apoptosis induced by miR-136-5p. The promotion of apoptosis was accompanied by upregulation of caspase-3 and Bax, and downregulation of Bcl-2, as estimated by western blotting. In conclusion, silencing of circ_RANBP9 inhibited GC proliferation and facilitated apoptosis by mediating the miR-136-5p/*XIAP* pathway. These findings provide a new theoretical basis for screening and treatment of PCOS.

## Introduction

Polycystic ovary syndrome (PCOS) is a widespread endocrine disease in women of childbearing age, and is characterized by hirsutism, anovulation, and polycystic ovaries [1]. According to epidemiological studies, up to 10% of women develop polycystic ovaries, worldwide, leading to abnormal reproductive functions, metabolic disorders, and an increased risk of diabetes and cardiovascular disease [2]. Because of the complexity of its pathogenesis, including heredity, environment, living habits, and diet, early screening is a challenging task [3]. PCOS is the cause of anovulatory infertility in 70% of women and about 30% of couples [4,5]. However, till date, no cure is available for this syndrome. Thus, novel targets for early diagnosis and treatment are required to alleviate the progression of PCOS.

Circular RNAs (circRNAs) are covalently closed continuous loops, the majority of which are conserved and stable in different species [6]. Accumulating evidence has revealed that circRNAs mediate gene expression through the regulation of transcription and translation by sponging RNA-binding proteins and/or microRNAs (miRNAs) [7]. circRNAs are involved in the pathogenesis of various diseases, such as cardiovascular diseases, neurologic diseases, and cancers in a tissue-specific manner [8–10]. Based on RNA sequencing (RNA-seq) data, abundant circRNAs were upregulated or downregulated in PCOS-affected women [11,12]. Dysregulation of circRNAs affects the incidence and progression of PCOS, leading to abnormal steroid production, adipocyte dysfunction, and alteration of ovarian cell proliferation and/or apoptosis [13]. circ_RANBP9 (hsa_circ_0001577), a non-coding RNA on chromosome 6, is upregulated in PCOS patients [11]. However, there are no studies on the functions of circ_RANBP9 in PCOS.

Thus, this study aimed to investigate the role of circ_RANBP9 in PCOS and explore its underlying mechanism. We found that a loss of circ_RANBP9 inhibited proliferation and facilitated apoptosis of ovarian granulosa cells (GCs). Furthermore, the miR-136-5p/*XIAP* axis was speculated be involved in the molecular mechanisms underlying the role of circ_RANBP9 in PCOS. Hence, this study may provide novel evidence for circ_RANBP9 as a therapy target of PCOS.

## Materials and methods

### Patients

A total of 90 participants, including 45 patients with PCOS and 45 healthy controls, were diagnosed in the Xiamen Key Laboratory of Genetic Testing (Xiamen, China). PCOS was diagnosed according to the Rotterdam standard. All the patients had not been previously diagnosed with PCOS (i.e., before attending this clinic) and had not received any treatment. Women with normal menstrual cycles and ovarian function served as controls. Plasma was isolated from blood (3 ml) of the participants under fasting conditions and stored in liquid nitrogen. All the participants provided written informed consent. This study was approved by the Ethics Committee of the Xiamen Key Laboratory of Genetic Testing (No. 201,801,005).

### Cell collection and culture

Human GCs (KGN and COV434) and human ovarian surface epithelial cells (IOSE80) were purchased from Chuanqiu Biotechnology (Shanghai, China). HEK293T cells were obtained from Biovector NTCC (Beijing, China). All the cells were maintained at 37°C under 5% CO_2_ in DMEM/F12 medium supplemented with 10% fetal bovine serum (FBS) and 1% penicillin/streptomycin (The reagents mentioned above were purchased from Hyclone, South Logan, UT, USA).

### Cell transfection

KGN and COV434 cells in the exponential growth phase were used for transfection when the cell confluence reached 75%. Empty vector, circ_RANBP9 expressing vector, siRNA (si)-negative control (si-nc: 5ʹ-CACUGAUUUCAAAUGGUGCUAUU-3ʹ), si-circ_RANBP9 1# (5ʹ-TAAACTTGGGTATAACTGT-3ʹ), si-circ_RANBP9 2# (5ʹ-TTGGGTATAACTGTTGTGT-3ʹ), miR-136-5p mimic (5ʹ-ACUCCAUUUGUUUUGAUGAUGGA-3ʹ)/inhibitor (5ʹ-UCCAUCAUCAAAACAAAUGGAGU-3ʹ), mimic/inhibitor nc (5ʹ-UCACAACCUCCUAGAAAGAGUAGA-3ʹ, 5ʹ-CAGUACUUUUGUGUAGUACAA-3ʹ), adenovirus (Ad)-nc, and Ad-*XIAP* were transfected into KGN and COV434 cells. Lipofectamine 2000 (Invitrogen, Carlsbad, CA, USA) was used for transient transfection of the cells. Next, the transfected cells were incubated for 48 h until the transfection efficiency was determined by Reverse Transcription-Quantitative Polymerase Chain Reaction (RT-qPCR).

### MTT assay

MTT assay was performed as previous described [14]. KGN and COV434 cells were seeded in 96-well plates. After incubation for 12, 24, 48, and 72 h, the cells were washed with PBS and incubated with 200 μl MTT solution for 2 h. Next, 150 μl of DMSO was added to the wells after discarding the supernatants. After 10 min, absorbance at 490 nm was measured using a microplate reader (BD Biosciences, San Jose, CA, USA).

### Colony formation assay

Cell proliferation was also assessed by colony formation assay [15]. Cells, post-transfection, were seeded in 6 cm cell culture dishes (Thermo Fisher Scientific, Waltham, MA, USA) and incubated at 37°C for 14 days. After discarding the medium, the cells were fixed with 4% paraformaldehyde and stained with 0.1% crystal violet. The cells were visualized under a microscope, and the numbers of colonies formed were counted in five random fields (>100 cells/each random field).

### Flow cytometry

Flow cytometry was conducted as previous described [16]. Cell apoptosis was assessed using the Annexin V PE/7-AAD apoptosis detection kit (Solarbio, Beijing, China). Briefly, the transfected cells were washed with PBS, and resuspended in 1ⅹ binding buffer to adjust the cell concentration to 2ⅹ10^6^ cells/ml. The cell suspension (100 μl) was incubated with 5 μl Annexin V/PE for 5 min in the dark. Next, 5 μl of 7-AAD solution at a concentration of 20 μg/ml was added, and the cells were washed with 400 μl PBS. Cell apoptosis rates were determined using flow cytometry on CytoFlex flow cytometer (Beckman Coulter, Miami, FL, USA).

### Luciferase reporter assay

3ʹUTRs of circ_RANBP9 and *XIAP* containing miR-136-5p binding sites were inserted into pmirGLO vectors (Promega, Madison, WI, USA). Plasmids with the target-site mutations were constructed (MUT circ_RANBP9 and MUT *XIAP*). HEK293T cells were seeded in 24-well plates. The cells were co-transfected with wild type (WT) plasmids or the corresponding MUT plasmids and miR-136-5p mimic or mimic NC using Lipofectamine 2000 (Invitrogen). Luciferase activity was analyzed using the Dual Luciferase Reporter Gene Assay Kit (Solarbio, Beijing, China) following the manufacturer’s instructions.

### RNA pull-down assay

Cells were transfected with biotinylated miR-136-5p (biotin-miR-136-5p) and matched biotinylated NC (biotin-NC). After 48 h, the cells were washed with PBS and incubated in a lysis buffer (Ambion, Austin, TX, USA) for 10 min. The lysates (50 μl) thus obtained were incubated with M‐280 streptavidin magnetic beads (Sigma–Aldrich, St. Louis, MO, USA). The Input group is lysates (50 μl) not incubated with without magnetic beads. Cells were incubated at 4°C for 3 h and washed with a washing buffer. Total RNA was isolated, and the levels of circ_RANBP9 or *XIAP* were measured using RT-qPCR.

### RT-qPCR

Total RNA was extracted using TRIzol reagent (Invitrogen) to detect the relative expression of circ_RANBP9, miR-136-5p, and *XIAP*. RNA (1 μg) was used for reverse transcription using PrimeScript™ RT Master Mix (Perfect Real Time) (Takara, Tokyo, Japan) and Mir-X miRNA First-Strand Synthesis Kit (Clontech, Mountain View, CA, USA). qPCR for circ_RANBP9 and *XIAP* was performed using TB Green® Premix Ex Taq™ (Tli RNaseH Plus) (Takara), while qPCR for miR-136-5p was performed using Mir-X miRNA qRT-PCR TB Green® Kit (Clontech). All the reaction were carried out according to the manufacturers’ instructions. GAPDH was used as an internal control for circ_RANBP9 and *XIAP*, while U6 was used for normalization of miR-136-5p. A fold change in relative expression was calculated using the 2^−ΔΔCt^ method. The primers used in this study were synthesized by GenScript (Nanjing, China). Specific primers sequences were as follows: circ_RANBP9 (F: 5ʹ-GGGCTTCAAACACCAGGAGA-3ʹ; R: 5ʹ-GTGCTTCCTTTGCCTGATGC-3ʹ), miR-136-5p (F: 5ʹ-ACTCCATTTGTTTTGATGATGGA-3ʹ; R: 5ʹ-CCAGTGCAGGGTCCGAGGT-3ʹ), XIAP (F: 5ʹ-CCGTGCGGTGCTTTAGTTGT-3ʹ; R: 5ʹ-TTCCTCGGGTATATGGTGTCTGAT-3ʹ), U6 (5ʹ-CTCGCTTCGGCAGCACA-3ʹ; 5ʹ-AACGCTTCACGAATTTGCGT-3ʹ) and GAPDH (F: 5ʹ-GAAAGCCTGCCGGTGACTAA-3ʹ; R: 5ʹ-GCGCCCAATACGACCAAATC-3ʹ).

### Western blot

Total protein was extracted using a protein extraction kit (KeyGEN BioTECH, Nanjing, China) to measure the expression of apoptosis-related factors. Protein concentrations were measured using the BCA kit (Sigma–Aldrich). Next, the proteins (30 μg) were separated using 10% SDS-PAGE and then transferred onto PVDF membranes (Merck Millipore, Billerica, MA, USA). After blocking with 5% skimmed milk, the membranes were incubated with primary antibodies at 4°C overnight, followed by incubation with secondary antibodies at room temperature for 1 h. Protein bands were developed using the ECL detection kit (KeyGEN BioTECH) and images were obtained. GAPDH was used as an internal control. The antibodies (Cell Signaling Technology, Danvers, MA, USA) used in this study were shown as follows: anti-XIAP (#2042, 1:1000), Caspase-3 (#9662, 1:1000), Bax (#2774, 1:1000), Bcl-2 (#3498, 1:1000) GAPDH (#2118, 1:1000), and HRP linked anti-Rabbit (#7074, 1:3000).

### Data analysis

Each experiment was replaced three times. All data in this study were analyzed using GraphPad Prism (Version 6; La Jolla, CA, USA). Measurement data are presented as mean ± SD. Comparisons among multiple groups were evaluated using one-way ANOVA, and Student’s t-test was used for comparison between two groups. P < 0.05 suggests a significant difference.

## Results

Here, we aimed to explore the role of circ_RANBP9 in PCOS. We conducted proliferation and apoptosis assays to evaluate the biological behaviors. We found a loss of circ_RANBP9 inhibited proliferation and facilitated apoptosis of GCs via regulating miR-136-5p/*XIAP* axis. Therefore, our data was the first to investigate the role of circ_RANBP9 and provide novel evidence for circ_RANBP9 as a therapy target of PCOS.

### Upregulation of circ_RANBP9 in patients with PCOS and GCs

**Figure 1.**
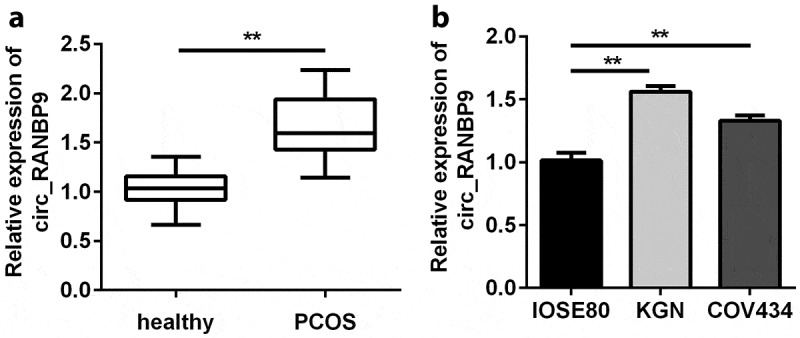
Circ_RANBP9 levels were elevated in patients with polycystic ovary syndrome (PCOS) and in ovarian granulosa cells (GCs). Different levels of circ_RANBP9 were compared in (a) plasma of patients with PCOS (n = 45) and healthy controls (n = 45), as well as (b) normal ovarian epithelium (IOSE80) cells and GCs (KGN, COV434). **P < 0.01

First, plasma samples obtained from 45 PCOS patients and 45 healthy controls were used to detect circ_RANBP9 levels. The expression of circ_RANBP9 in the plasma of PCOS patients was significantly higher than that in controls (P < 0.01; Figure 1a). These results are consistent with those obtained for GCs. As shown in Figure 1b, circ_RANBP9 was significantly elevated in KGN and COV434 cells than in IOSE80 cells (P < 0.01).

### Loss of circ_RANBP9 suppressed proliferation and accelerated apoptosis of GCs

**Figure 2.**
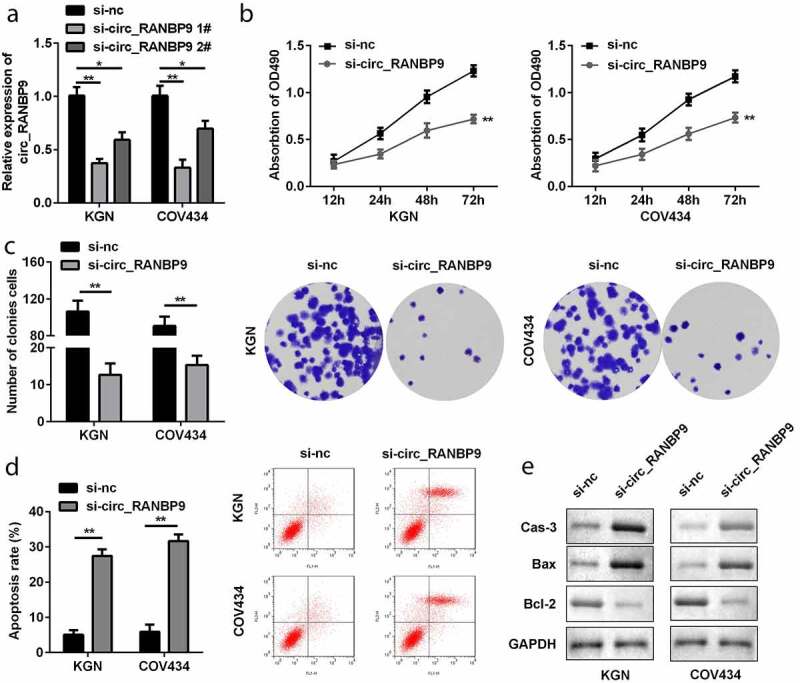
Effects of circ_RANBP9 on proliferation and apoptosis of GCs. (a) circ_RANBP9 levels were tested by RT-qPCR. (b) MTT assay was conducted to determine the proliferation of GCs. (c) Colony formation assay was performed to detect the proliferation of GCs. (d) Flow cytometry was carried out to measure the apoptosis rates of GCs. (e) Protein levels of caspase-3, Bax, and Bcl-2 via western blot analyses were determined post-transfection. **P < 0.01

To explore the function of circ_RANBP9, its level was modulated in GCs by transfection with si-nc and si-circ_RANBP9. Downregulation of circ_RANBP9 was observed in the si-circ_RANBP9 1# (P < 0.01) and si-circ_RANBP9 2# groups (P < 0.05), particular in si-circ_RANBP9 1# group (Figure 2a). si-circ_RANBP9 1# was used in the following experiment. Functionally, knockdown of circRANBP9 significantly suppressed the proliferation of GCs (P < 0.01; Figure 2b and c). By contrast, apoptosis of GCs was significantly promoted in the si-circ_RANBP9 than in the si-nc group (P < 0.01; Figure 2d). Moreover, knockdown of circ_RANBP9 enhanced caspase-3 and Bax levels, but repressed Bcl-2 levels (Figure 2e).

### Circ_RANBP9 is a sponge of miR-136-5p

**Figure 3.**
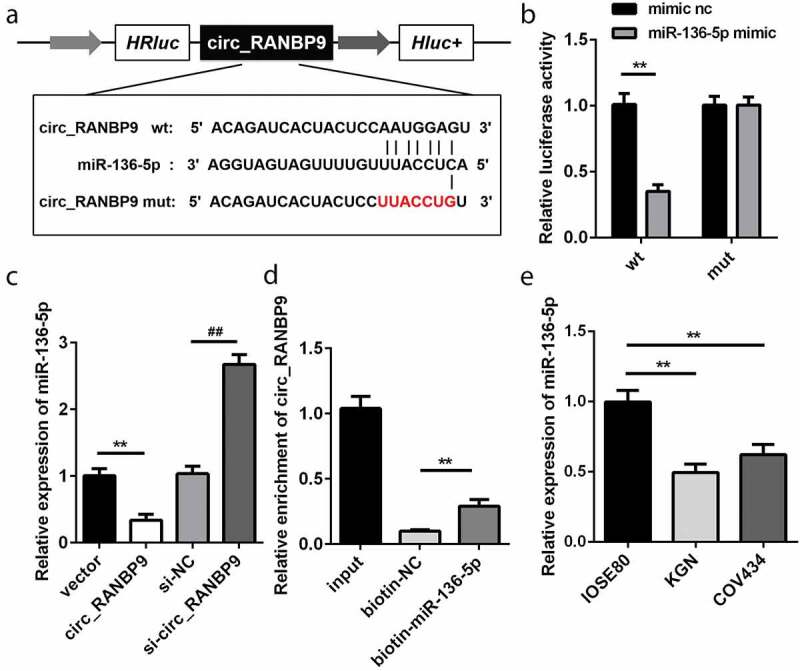
miR-136-5p was negatively regulated by circ_RANBP9. (a) Direct binding sites of miR-136-5p in WT 3ʹ-UTR of circ_RANBP9 and MUT 3ʹ-UTR of circ_RANBP9 were designed. (b) WT circ_RANBP9 or MUT circ_RANBP9 recombinant plasmids and miR-136-5p mimic or mimic nc were co-transfected and luciferase reporter assay was performed. (c) miR-136-5p levels determined using RT-qPCR were detected in GC overexpression or knockdown of circ_RANBP9. (d) Interaction between miR-136-5p and circ_RANBP9 was confirmed by RNA pull-down. (e) Expression of miR-136-5p in normal ovarian epithelium (IOSE80) cells and GCs (KGN, COV434) was detected by RT-qPCR. **P < 0.01. ^##^P < 0.01

Based on bioinformatics analysis, the WT 3ʹ-UTR of circ_RANBP9 rather than MUT 3ʹ-UTR of circ_RANBP9 was able to partly bind to miR-136-5p (Figure 3a). As compared to the transfection of mimic nc, that of miR-136-5p mimic decreased luciferase activity in HEK293T cells expressing the WT 3ʹ-UTR of circ_RANBP9 (P < 0.01). However, luciferase activity did not influence the cells that expressed MUT 3ʹ-UTR of circ_RANBP9 (Figure 3b). miR-136-5p levels were significantly decreased by circ_RANBP9 (P < 0.01) and were significantly increased by knockdown of circ_RANBP9 (P < 0.01; Figure 3c). The interaction between circ_RANBP9 and miR-136-5p was further confirmed by an RNA pull-down assay (P < 0.01; Figure 3d). The level of miR-136-5p was significantly reduced in the GCs of KGN and COV434 cells than in those of IOSE80 cells (P < 0.01; Figure 3e).

### Loss of circ_RANBP9 targeting miR-136-5p suppressed proliferation and accelerated apoptosis of GCs

**Figure 4.**
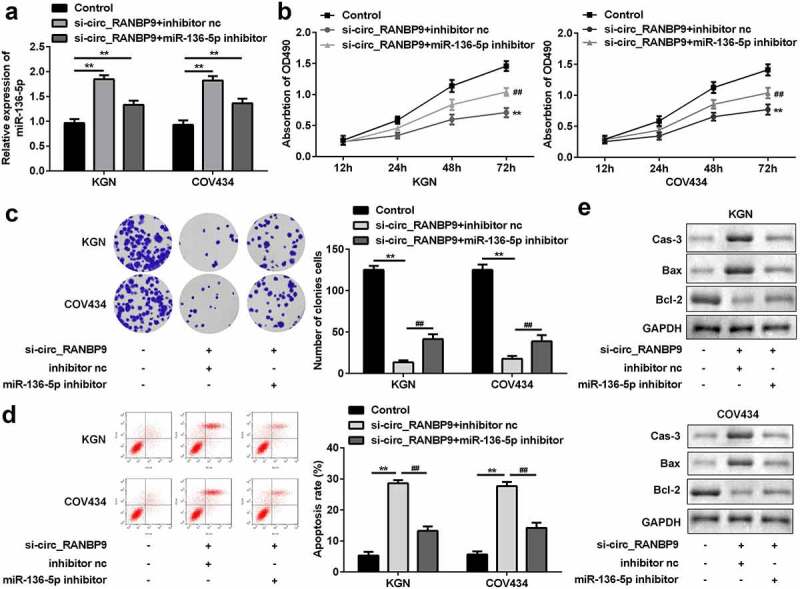
Inhibition of miR-136-5p abolished the effects on proliferation and apoptosis induced by knockdown of circ_RANBP9. (a) Expression of miR-136-5p was tested in transfected cells by RT-qPCR. Cell proliferation was assessed post-transfection by (b) MTT assay and (c) colony formation assay. (d) Flow cytometry was performed to detect apoptosis rates of GCs. (e) Protein levels of caspase-3, Bax, and Bcl-2 at was determined by western blotting. **P < 0.01. ^##^P < 0.01

To investigate the underlying molecular mechanisms, the level of miR-136-5p was estimated in GCs transfected with si-circ_RANBP9 and miR-136-5p inhibitor. The expression of miR-136-5p was upregulated by the knockdown of circ_RANBP9 and was rescued by the inhibitor (P < 0.01; Figure 4a). Silencing of circ_RANBP9 inhibited cell proliferation, while the miR-136-5p inhibitor reversed these effects (P < 0.01; Figure 4b and c). By contrast, cell apoptosis was accelerated by circ_RANBP9 knockdown, which was abolished by miR-136-5p inhibition (P < 0.01; Figure 4d). Regarding apoptosis-related factors, si-circ_RANBP9 significantly enhanced caspase-3 and Bax levels but decreased Bcl-2 levels, while miR-136-5p inhibitor abolished the effects induced by si-circ_RANBP9 (Figure 4e).

### XIAP is a target of miR-136-5p

**Figure 5.**
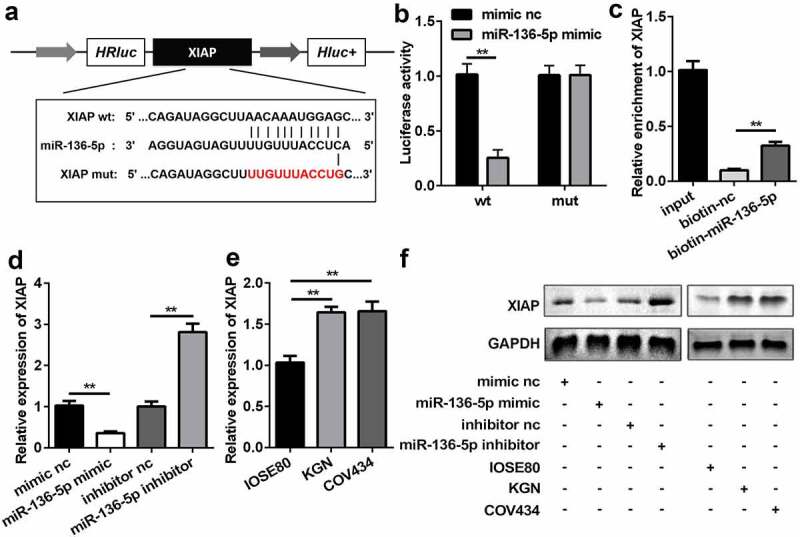
miR-136-5p directly targeted downstream *XIAP*. (a) Direct binding sites between miR-136-5p and WT 3ʹ-UTR of *XIAP* are shown. MUT 3ʹ-UTR of circ_RANBP9 was also designed. (b) Luciferase activity was tested in HEK293T cells transfected by WT *XIAP* or MUT *XIAP* recombinant plasmids and miR-136-5p mimic or mimic nc. (c) Interaction between *XIAP* and miR-136-5p was assessed using RNA pull-down assay. (d) *XIAP* expression was determined by RT-qPCR in overexpression or inhibition of miR-136-5p cells. (e) Comparison of *XIAP* using RT-qPCR in IOSE80 cells and GCs (KGN, COV434). (f) The expression of *XIAP* was measured using western blotting. **P < 0.01. ^##^P < 0.01

The results of bioinformatics analysis showed that *XIAP* had possible binding sites for miR-136-5p (Figure 5a). HEK293T cells co-transfected with miR-136-5p mimic and WT 3ʹ-UTR of *XIAP* significantly reduced luciferase activity (P < 0.01), which was not affected in the MUT group (Figure 5b). Additionally, the RNA pull-down assay further verified the interaction between *XIAP* and miR-136-5p (P < 0.01; Figure 5c). Overexpression of miR-136-5p significantly repressed the expression of *XIAP*, whereas inhibition of miR-136-5p enhanced the expression of *XIAP* (P < 0.01; Figure 5d and f). The levels of *XIAP* were higher in KGN and COV434 cells than in IOSE80 cells (Figure 5e and f).

### miR-136-5p targets XIAP to influence proliferation and apoptosis of GCs

**Figure 6.**
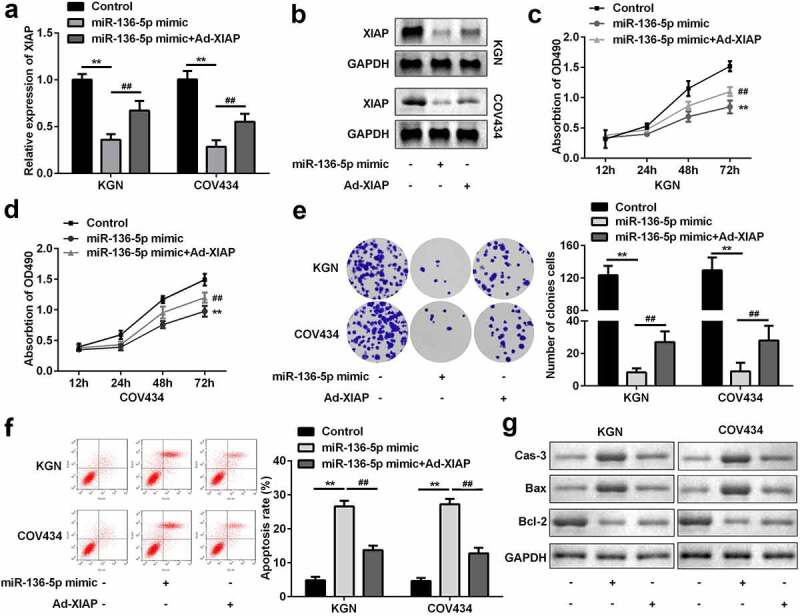
*XIAP* reversed the effects on proliferation and apoptosis induced by miR-136-5p. (a) *XIAP* levels were measured by RT-qPCR. (b) *XIAP* levels were detected by western blotting. Cell proliferation by (c) MTT assay and (d) colony formation assay performed post-transfection is shown. (e) Cell apoptosis was determined by flow cytometry. (f) Protein levels of caspase-3, Bax, and Bcl-2 were determined using western blotting. **P < 0.01. ^##^P < 0.01

To further explore the mechanism, miR-136-5p mimic and Ad-*XIAP* were transfected into GCs. The level of *XIAP* was significantly downregulated by overexpression of miR-136-5p, which was reversed by overexpression of *XIAP* (P < 0.01; Figure 6a and b). Functionally, overexpression of miR-136-5p significantly inhibited the proliferation of GCs; however, upregulation of *XIAP* abolished this inhibition (P < 0.01; Figure 6c–e). Moreover, upregulation of *XIAP* significantly suppressed the effects of miR-136-5p and suppressed apoptosis of GCs (P < 0.01; Figure 6f). As illustrated in Figure 6g, the upregulation of caspase-3 and Bax, as well as the downregulation of Bcl-2 were induced by miR-136-5p, while *XIAP* rescued these effects.

## Discussion

Growing evidence indicates that circRNAs are involved in the incidence and development of PCOS [13]. However, the pathogenesis of PCOS is still not fully understood. Excessive proliferation of granulosa cells is one of the main causes of ovarian dysfunction, which contributes to follicular atresia and further induces PCOS [17]. It is well known that circRNAs are collectively involved in the initiation and development of PCOS. For example, circPSMC3 suppresses granulosa tumor cell proliferation and facilitates their apoptosis in PCOS [18]. Additionally, downregulation of circPUM1 enhances proliferation and inhibits apoptosis [19]. Besides, circASPH knockdown represses proliferation and accelerates apoptosis of KGN cells [20]. However, there are no reports on the role of circ_RANBP9 (hsa_circ_0001577) in PCOS. circ_RANBP9, a circRNA derived from the RANBP9 gene, codes for the scaffolding protein RANBP9, which exists in the cytoplasm and nucleus and is associated with cellular processes [21]. A previous study revealed that hsa_circ_0001577 is a candidate circRNA for colorectal cancer [22]. Additionally, circ_RANBP9 levels were found to be increased in granulosa cells of patients with PCOS [11]. In the current study, we found that circ_RANBP9 levels were elevated in the plasma of patients with PCOS and granulosa cells KGN and COV434. Knockdown of circ_RANBP9 decelerates cell proliferation and promotes apoptosis. Therefore, knockdown of circ_RANBP9 may be a promising therapy for the treatment of PCOS. However, the underlying mechanisms of action require further study.

circRNAs act as competing endogenous RNAs (ceRNAs) and regulate biological processes by sponging miRNAs [7]. In this study, miR-136-5p was the target of circ_RANBP9. miR-136-5p is involved in the progression of human cancers, such as thyroid carcinoma [23], hepatocellular carcinoma [24], and renal cell carcinoma [25]. In gynecological diseases, miR-136-5p is regulated by long noncoding RNA (lncRNA) FAM83H-AS1 to suppress cell proliferation, invasion, and migration of breast cancer cells [26]. Similarly, miR-136-5p is also involved in the proliferation, migration, and invasion of cervical cancer cells [27]. In PCOS, miR-136 levels are decreased, which reverse the effects of circ_0118530 on cell survival rate, apoptosis, migration, and oxidative stress in KGN cells [28]. In our study, miR-136-5p was found to be downregulated in PCOS. Moreover, circ_RANBP9 sponged miR-136-5p to modulate the proliferation and apoptosis of GCs. These findings suggest that loss of circ_RANBP9 inhibits proliferation and facilitates apoptosis of GCs by sponging miR-136-5p.

*XIAP* is a member of the inhibitor of apoptosis protein (IAP) family and has an inhibitory effect on the activation of caspases 3, 7, and 9 [29]. *XIAP* is also involved in the development of cells and signaling pathways [30]. *XIAP* is widely expressed in the ovary, especially in thecal cells and granulosa cells [31]. *XIAP* has been reported to be upregulated in PCOS [30]. However, inhibition of *XIAP* and restoration of the PPARγ pathway could suppress proliferation and induce apoptosis of KGN cells [32]. In the current study, *XIAP* was upregulated in GCs; this result was consistent with previous research [33] and *XIAP* was confirmed to be a target of miR-136-5p. *XIAP* abolished the inhibition of proliferation and enhanced apoptosis induced by miR-136-5p. Taken together, these results suggest that miR-136-5p targets *XIAP* to repress GC cell proliferation and accelerate apoptosis.

During the process of apoptosis, Bax and Bcl-2 are two members of the Bcl-2 family possessing pro-apoptotic and anti-apoptotic effects, respectively [34]. Caspase-3 is also activated during apoptosis [35]. Therefore, the facilitation of apoptosis was accompanied by upregulation of caspase-3 and Bax; however, Bcl-2 levels were repressed in our study.

## Conclusion

The expression of circ_RANBP9 was upregulated in patients with PCOS and in GCs. miR-136-5p was downregulated, and *XIAP* was upregulated in GCs. Furthermore, knockdown of circ_RANBP9 suppressed proliferation and facilitated apoptosis of GCs through the miR-136-5p/*XIAP* axis. This might provide a novel theoretical basis for the screening and treatment of PCOS.

## Research highlights

1. circ_RANBP9 is overexpressed in polycystic ovary syndrome.
2. Knockdown of circ_RANBP9 inhibits the proliferation and promotes apoptosis of GCs.
3. circ_RANBP9 sponges miR-136-5p to regulate *XIAP*.
4. Knockdown of circ_RANBP9 alleviates PCOS progression by miR-136-5p/*XIAP* axis.
